# Clinical role, safety and diagnostic accuracy of percutaneous transthoracic needle biopsy in the evaluation of pulmonary consolidation

**DOI:** 10.1186/s12931-019-0982-5

**Published:** 2019-01-31

**Authors:** Nantaka Kiranantawat, Shaunagh McDermott, Florian J. Fintelmann, Sydney B. Montesi, Melissa C. Price, Subba R. Digumarthy, Amita Sharma

**Affiliations:** 1 Division of Thoracic Imaging and Intervention, Department of Radiology, Massachusetts General Hospital, 55 Fruit St, Boston, MA 02114 USA; 2 Department of Radiology, Songklanagarind Hospital, Prince of Songkhla University Hat Yai, Songkhla, 90110 Thailand; 3 Pulmonary and Critical Care Medicine, Massachusetts General Hospital, Boston, MA USA

**Keywords:** Percutaneous transthoracic needle biopsy, Consolidation, Pneumothorax, Hemoptysis

## Abstract

**Background:**

To determine the clinical role, safety, and diagnostic accuracy of percutaneous transthoracic needle biopsy in the evaluation of pulmonary consolidation.

**Methods:**

A retrospective review of all computed tomography (CT)-guided percutaneous transthoracic needle biopsies (PTNB) at a tertiary care hospital over a 4-year period was performed to identify all cases of PTNB performed for pulmonary consolidation. For each case, CT Chest images were reviewed by two thoracic radiologists. Histopathologic and microbiologic results were obtained and clinical follow-up was performed.

**Results:**

Thirty of 1090 (M:F 17:30, mean age 67 years) patients underwent PTNB for pulmonary consolidation (2.8% of all biopsies). A final diagnosis was confirmed in 29 patients through surgical resection, microbiology, or clinicoradiologic follow-up for at least 18 months after biopsy. PTNB had an overall diagnostic accuracy of 83%. A final diagnosis of malignancy was made in 20/29 patients, of which 19 were correctly diagnosed by PTNB, resulting in a sensitivity of 95% and specificity of 100% for malignancy. In all cases of primary lung cancer, adequate tissue for molecular testing was obtained. A benign final diagnosis was made in 9 patients, infection in 5 cases and non-infectious benign etiology in 4 cases. PTNB correctly diagnosed all cases of infection. Minor complications occurred in 13% (4/30) of patients.

**Conclusions:**

Pulmonary consolidation can be safely evaluated with CT-guided percutaneous needle biopsy. Diagnostic yield is high, especially for malignancy. PTNB of pulmonary consolidation should be considered following non-diagnostic bronchoscopy.

## Introduction

Pulmonary consolidation is a frequently encountered clinical entity which is most commonly due to an acute infection. In a small proportion of cases, pulmonary consolidation fails to resolve. Consolidation is considered to be persistent when the opacity has failed to resolve by 50% in 2 weeks or completely in 4 weeks [1]. Persistent consolidation may be due to inadequately treated or atypical infections, malignancy, organizing pneumonia, chronic eosinophilic pneumonia, sarcoidosis or vasculitis [2]. This broad differential creates a clinical dilemma that frequently necessitates further testing including bronchoscopy and tissue sampling.

While no consensus guidelines exist, bronchoscopy with bronchoalveolar lavage and often transbronchial biopsy is usually the preferred method for evaluation of non-resolving pulmonary consolidation [3]. In cases where bronchoscopic evaluation is non-diagnostic, additional tissue sampling is indicated. Traditionally, surgical biopsy was performed in such cases; however, CT-guided percutaneous needle biopsy (PTNB) is an appealing alternative as it is less invasive and associated with fewer complications.

PTNB is a well-established, safe technique for the diagnosis of pulmonary nodules or masses [4–6], and the role of PTNB for obtaining tissue for molecular testing of lung cancer continues to grow [7–9]. There are few studies reporting the diagnostic accuracy and safety of PTNB in pulmonary consolidation [10, 11], and no study assessing tissue yield for molecular testing in the setting of primary lung cancer presenting as consolidation. Therefore, we aimed to determine the clinical role, safety and diagnostic accuracy of CT-guided PTNB in the evaluation of persistent pulmonary consolidation and to identify risk factors for diagnostic failure.

## Materials and methods

The study was a retrospective single-center study and performed with IRB approval from the Partners Human Research Committee (Project number 2014P001409). Due to the retrospective design, the institutional ethical review board waived the need for informed consent. The study was designed and conducted according to Standards for Reporting of Diagnostic Accuracy (STARD) [12]. Complications were categorized in accordance with the Society of Interventional Radiology clinical practice guidelines [13].

### Biopsy procedure

All biopsies were performed by experienced thoracic radiologists according to a standard protocol [14]. The procedures were performed under CT guidance using a 16-slice multidetector CT (MDCT) scanner (General Electric, Fairfield, CT). Patients were positioned prone, or supine, depending on the location of the lesion. The needle trajectory was planned using 2.5-mm thick slices to avoid bullae, emphysematous lung and fissures, so as to cross the fewest pleural layers. If needed, the CT gantry was angled to avoid vessels, fissures and ribs. First, a 19-guage thin-walled coaxial introducer needle (Chiba-Ultrathin, Cook) was advanced into the lesion. Whenever possible, the densest part of the consolidation was targeted, rather than areas of ground glass, with avoidance of large vessels or air bronchograms. Intermittent limited CT scans were used for evaluating needle trajectory. The pleura was punctured only once during the procedure to minimize the risk of pneumothorax. Once CT confirmed that the needle was in the lesion, the inner stylet was removed and a 22-gauge Chiba aspiration needle was advanced through the lumen of the introducer needle. Fine needle aspirates (FNA) were obtained and submitted to the on-site cytopathologist for rapid interpretation. Microbiology was submitted when this on-site evaluation was either negative for malignancy or suspicious for infection. Whenever appropriate and safe, core needle biopsy (CNB) specimens were also obtained through the introducer needle with a spring-loaded 20-gauge biopsy device (Cook Medical, Bloomington, IN) with a 1- or 2-cm needle throw.

### Medical record review and image analysis

A search of the radiology database was performed to identify patients who had undergone CT-guided PTNB of pulmonary consolidation between July 2009 and June 2013. The patient’s electronic medical record was reviewed to identify the indication for biopsy. Any imaging preceding the biopsy was recorded and reviewed to confirm the persistence of consolidation. Previous attempts at tissue sampling through alternative methods were recorded. The decision to biopsy was based on the size and location of the lesion, and exclusion of contraindications, such as bleeding diatheses.

Two board-certified radiologists (NK and SM, 4 and 6 years of experience, respectively) reviewed procedural images of all cases on a clinical workstation (Impax 4.1, Agfa HealthCare, Antwerp, Belgium) to determine extent and location of the consolidation. Images and procedure reports were reviewed to determine development of any complications including pneumothorax during or after the procedure, chest tube placement, hemoptysis and procedure-related mortality.

### Classification of biopsy result and final diagnosis

The histopathologic reports of PTNB were divided into four categories: malignant or suspicious for malignancy, specific benign, non-specific benign, and non-diagnostic. Malignant or suspicious for malignancy included cases in which the pathologic report described specific malignant tumor or findings suspicious for malignancy. Specific benign included cases in which a benign neoplasm such as hamartoma or specific infection was diagnosed. A sample was regarded as non-specific benign if pathological findings such as fibrosis, inflammation without identification of specific microorganisms, and necrosis were present but a specific disease could not be diagnosed. If specimens contained only normal respiratory elements such as lung tissue, respiratory epithelial cells, histiocytes, or blood, the sample was considered non-diagnostic. The final diagnosis was established through biopsy result, surgical correlation, microbiology or clinicoradiologic follow-up for at least 18 months following biopsy.

The overall accuracy of PTNB for a specific diagnosis of malignancy, infection or a benign etiology was calculated, as well as sensitivity and specificity of PTNB for the diagnosis of malignancy. A PTNB result of malignant or suspicious for malignancy was considered to be a positive result. A positive result was considered to be true-positive when there was surgical or clinical confirmation. Specific benign, non-specific benign and non-diagnostic PTNB diagnoses were considered to be negative results. A negative biopsy result was considered to be true-negative when a benign neoplasm or specific infection was diagnosed, or when the clinical diagnosis of a benign etiology was made. If surgical pathology or clinic-radiologic follow-up resulted in a diagnosis of malignancy, the PTNB result was considered to be false-negative.

The sensitivity and specificity of PTNB for infection were calculated. A positive result was considered to be a true-positive when there was a specific infectious organism identified by PTNB and the patient had a response to subsequent treatment. If there was no clinical evidence of infection or the lesion demonstrated regression at follow-up CT without therapy, a positive PTNB result for infection was considered to be a false-positive. Malignant or suspicious for malignancy, non-specific benign and non-diagnostic were considered to be a negative result for the diagnosis of infection. A negative result was considered to be a true-negative when a clinical diagnosis of non-infectious disease was made. If surgical resection or clinical evidence was suggestive of infection, the PTNB result was considered to be a false-negative.

To determine risk factors for diagnostic failure of PTNB, patients were classified into two groups: the diagnostic success group and the diagnostic failure group. The diagnostic success group included truly diagnosed cases. The diagnostic failure group included non-diagnostic, non-specific benign and falsely diagnosed cases.

### Statistical analysis

Statistical differences between two groups were analyzed by using the student’s t test for continuous variables of patient age and needle path length within the lung. Statistical differences in patient, lesion and procedure characteristics were analyzed by using the Fisher exact test. All statistical testing was performed using statistical software (Stata, version 14.2, StataCorp) with *p* value of 0.05 or less considered significant.

## Results

Over a four-year period, 1090 CT-guided percutaneous transthoracic needle biopsies (PTNB) were performed. Thirty (2.8%) were performed for diagnostic evaluation of consolidation. The majority of patients were male, were former or current smokers, and had a mean age of 67. Half of the patients had a history of lung cancer. In 3 cases the preceding imaging was performed in an outside hospital and was not available for review. Of the remaining cases, CT was the most common imaging modality with only 26% having a PET scan prior to biopsy. Only 5 (19%) had one cross-sectional imaging study prior to biopsy. Nine (33%) had between 2 and 5 studies, four (15%) had between 6 and 10 studies, and the remaining 9 (33%) had greater than 10. The biopsy was performed to rule out infection in 9 cases, to exclude malignancy in 9 cases and to provide material for molecular testing of lung cancer in 12 cases. The characteristics of the patients are summarized in Table 1.

**Fig. 1:**
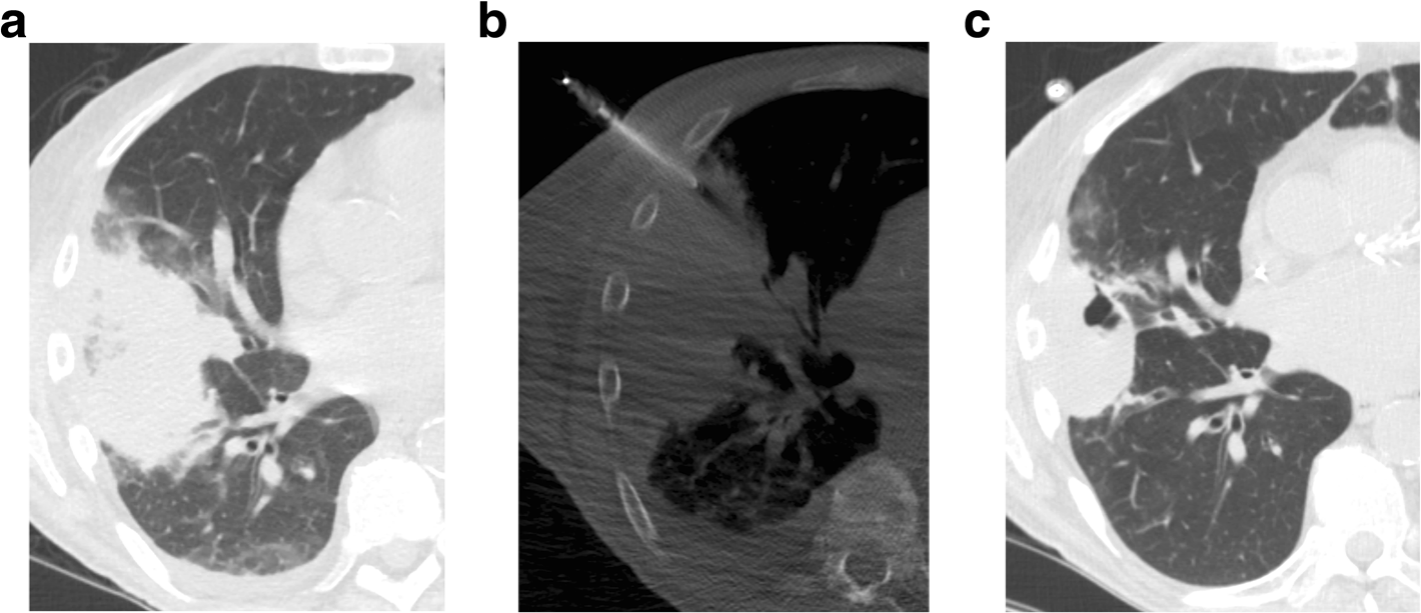
**a**) Axial CT scan in a 69-year-old man status post kidney transplant demonstrates an area of consolidation and surrounding ground glass opacity in the right middle and lower lobe. He underwent bronchoalveolar lavage and transbronchial biopsy which found acute hemorrhage and hemosiderosis. **b**) Axial CT scan obtained during PTNB, performed 2 days after bronchoscopy, shows the needle within the consolidation in the right middle lobe. Fungal hyphae were identified on cytologic evaluation and aspergillus was identified on accompanying microbiology culture. **c**) Axial CT performed three months later, on treatment with ambisome, shows the area of consolidation improving

**Table 1:** Demographics and lesion characteristics of patients undergoing percutaneous transthoracic needle biopsy for persistent pulmonary consolidation

Characteristics	No (%)
Age, yr. (mean ± SD)	67 ± 10
Sex	
Male	17 (57)
Female	13 (43)
Smoking history	
Non-smoker	8 (27)
Current smoker	4 (13)
Former smoker	18 (60)
Bronchoscopy prior to PTNB	
No	23 (77)
Yes	7 (23)
Underlying disease	
None	6 (20)
Lung cancer	15 (50)
Other malignancy	4 (13)
Leukemia	1 (3)
Lymphoma	1 (3)
Pancreatic cancer	1 (3)
Breast cancer	1 (3)
Immunocompromised	1 (3)
Pulmonary disease	4 (13)
Indication for biopsy	
R/O Infection	9 (30)
R/O Cancer	9 (30)
Molecular testing in known lung cancer	12 (40)
Treatment before biopsy	
None	23 (77)
Antibiotic	5 (17)
Steroid	1 (3)
Antibiotic and Steroid	1 (3)
Chemotherapy and/or targeted therapy	15 (50)
Emphysema	
Absent	20 (67)
Present	10 (33)
Lobar involvement of consolidation	
< 1/2 Lobe	10 (33)
> 1/2 Lobe	5 (17)
> Lobe	15 (50)
Distribution	
Single lung	18 (60)
Bilateral	12 (40)
Location of biopsied consolidation	
Right upper lobe	6 (20)
Right middle lobe	2 (7)
Right lower lobe	7 (23)
Left upper lobe	10 (33)
Left lower lobe	5 (17)
Ground glass component	
Absent	10 (33)
Present	20 (67)
Cavitation	
Absent	26 (87)
Present	4 (13)
Hilar adenopathy	
Absent	21 (70)
Present	9 (30)
Mediastinal adenopathy	
Absent	22 (73)
Present	8 (24)
Pleural effusion	
Absent	17 (57)
Present	13 (43)

The consolidation involved a single lung in 60% of cases. There was an associated ground glass component in 67% of cases. Cavitation was present in 4 cases. The upper lobes were the most common location biopsied. The characteristics of the biopsied lesions are also summarized in Table 1.

**Fig. 2:**
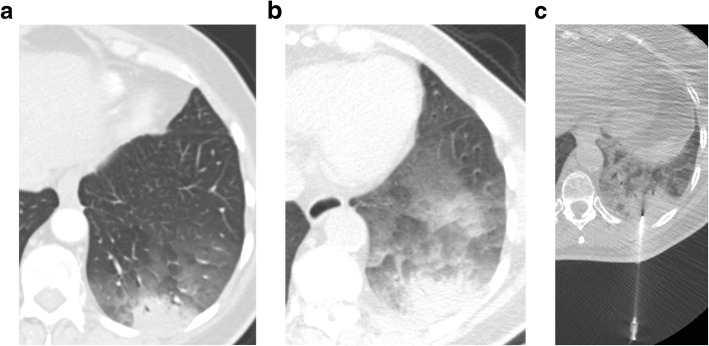
**a**) Axial CT scan in a 74-year-old woman demonstrates consolidation and ground glass opacity in the left lower lobe. **b**) Axial CT scan performed 3 months later demonstrates persistent consolidation and increased ground glass opacity in the left lower lobe. She underwent bronchoalveolar lavage and transbronchial biopsy which found bronchial columnar cells and macrophages but no evidence of malignancy. **c**) Axial CT scan obtained during PTNB, performed 4 weeks after the bronchoscopy shows the needle within the consolidation in the left lower lobe. Well-differentiated lung adenocarcinoma was confirmed on histology

Of the 30 patients, PTNB accurately established a diagnosis in 83% of cases as summarized in Table 2. A final diagnosis of malignancy was established in 20 cases. PTNB demonstrated a sensitivity of 95% and specificity of 100% for malignancy. FNA and CNB failed to make the diagnosis in 1 case of lung cancer, which was made on surgical resection. 14/20 cases of confirmed malignancy were lung cancer, and in all 14 cases CNB [12] and FNA [2] were adequate for molecular testing. A benign etiology was established in 9 cases. PTNB demonstrated 100% sensitivity (5/5 cases) for the diagnosis of infection, even in three cases where the patient was receiving antibiotics prior to biopsy. However, the overall sensitivity for the diagnosis of benignity was only 55%, as the biopsy results of the non-infectious benign cases were either non-specific benign or non-diagnostic.

**Table 2:** Association between diagnosis obtained from percutaneous transthoracic needle biopsy and final diagnosis (*N* = 29)

Lesion	PTNB	Final Diagnosis
*Malignant lesions*		
Lung cancer	16	17
Lymphoma	1	1
Leukemia	1	1
Metastatic pancreatic cancer	1	1
*Benign lesions*		
Mycobacterium avium intracellulare (MAI) infection	2	2
Bacterial infection	2	2
Fungal infection	1	1
Post radiation change	0	2
Non-specific benign (Scar/Inflammation/Fibrosis)	3	2
*Non-diagnostic*	2	0

Of the patients who underwent biopsy for suspected infection, infection was confirmed in 56% (5/9), malignancy was diagnosed in 22% (2/9)(chronic lymphocytic leukemia (CLL) and metastatic pancreatic cancer), one patient had stable follow-up imaging for more than 2 years indicating benignity and one patient was lost to follow-up.

PTNB diagnosed the etiology of consolidation in all 5 subjects who had previously undergone a non-diagnostic fiberoptic bronchoscopy (Table 3). The reason for two additional subjects to undergo PTNB after bronchoscopy was the need for additional tissue for molecular testing of known lung cancer.

**Table 3:** Association between diagnosis obtained from percutaneous transthoracic needle biopsy and bronchoscopy

Percutaneous Transthoracic Needle Biopsy	Bronchoscopy
Lung cancer (*n* = 4)	Lung cancer (*n* = 2) Negative for malignancy (*n* = 2)
Chronic lymphocytic leukemia (*n* = 1)	Bacterial infection
Bacterial infection (*n* = 1)	Acute inflammation
Fungal infection (*n* = 1)	Hemorrhage

There were 6 cases of diagnostic failure, including 4 cases of non-specific benign and 2 cases of non-diagnostic sample. The relationship between the two groups is shown in Table 4. Lesions without ground-glass component were significantly associated with diagnostic failure (*p* = 0.009). Other patient, lesion or procedure characteristics were not significantly different between the two groups. Final diagnoses were made in 5 cases, with one patient lost to follow-up. In one case, in which both FNA and CNB was obtained, the final diagnosis was lung cancer. In the four remaining cases, the lesions were stable for more than 2 years on imaging, indicating benignity. A final diagnosis of benignity was significantly higher in the diagnostic failure group (*p* = 0.02). There was no significant difference in the rate of diagnostic failure if FNA and CNB, or FNA alone was obtained.

**Table 4:** Characteristics of patients and lesions in diagnostic success and failure groups

Variable	Success (*n* = 24)	Failure (*n* = 6)	*P* value
*Patient characteristics*			
Age, yr. (mean ± SD)	66 ± 10	72 ± 10	0.2
Sex			0.2
Male	12	12	
Female	12	12	
History of malignancy			0.6
No	9	2	
Yes	15	4	
Clinical suspicion			1
Infection	7	2	
Cancer	17	4	
Bronchoscopy before biopsy			0.3
No	17	6	
Yes	7	0	
*CT findings*			
Emphysema			0.1
Absent	18	2	
Present	6	4	
Distribution of consolidation			0.7
Single lung	13	4	
Bilateral	11	2	
Ground glass component			0.009
Absent	5	5	
Present	19	1	
Presence of cavitation			0.6
Absent	20	6	
Present	4	0	
Hilar adenopathy			0.6
Absent	16	5	
Present	8	1	
Mediastinal adenopathy			1
Absent	17	5	
Present	7	1	
Pleural effusion			0.2
Absent	12	5	
Present	12	1	
*Procedure Characteristics*			
Biopsy site			0.07
Above the hilum	9	5	
Below the hilum	15	1	
Transversed aerated lung			1
No	21	5	
Yes	3	1	
Area biopsied			0.7
Edge	13	4	
Center	11	2	
Needle path, mm (mean ± SD)	17 ± 1	14 ± 1	0.5
Hemoptysis			1
No	22	6	
Yes	2	0	
Pneumothorax			0.5
No	22	5	
Yes	2	1	
Samples obtained			1
FNA only	7	1	
FNA and core	17	5	
*Final diagnosis*^a^			0.02
Malignant	19	1	
Benign	5	4	

^a^final diagnosis was only available for 29 patients as one patient was lost to follow-up

Student t-test was used for continuous variables

Fisher exact test was used for categorical variables

There were no deaths or major complications resulting from PTNB. Minor complications occurred in 4/30 patients (13%), including 3 pneumothoraces identified on post-biopsy chest radiograph, one of which required chest tube placement, and 2 cases of self-limited hemoptysis, one of which occurred in a patient with a small pneumothorax who was hospitalized overnight for observation.

## Discussion

In our study, the diagnostic accuracy of PTNB for consolidation was 83%. PTNB had a sensitivity and specificity for malignancy of 95 and 100%, respectively, and a 100% sensitivity for the diagnosis of infection. Diagnostic failure of PTNB occurred in 20% of cases; 4 cases reported as non-specific benign and 2 cases reported as a non-diagnostic sample. However, only one of these patients went on to be diagnosed with primary lung cancer. A final diagnosis of benignity was significantly higher in the diagnostic failure group than the diagnostic success group. Consolidation without a ground-glass component was associated with diagnostic failure. This may be explained by the ground glass component reflecting an active inflammatory etiology or more aggressive malignancy. Our study did not observe greater diagnostic failure or complications in the presence of emphysema or lesion cavitation. There was also no statistically significant difference in diagnostic failure rates when the distance of lung traversed was higher or when aerated lung was crossed as has been reported in previous studies [15, 16].

Persistent consolidation is a rare indication for PTNB in clinical practice and initial evaluation is typically performed with bronchoscopy and bronchoalveolar lavage, often along with transbronchial biopsy. A study in immunocompromised patients reported that fiberoptic bronchoscopy was more likely to make the final diagnosis when consolidation was due to an infectious process (81%), while the diagnostic yield for noninfectious processes was only 56% [17]. We found that CT-guided PTNB provided definite diagnoses in all cases of consolidation, both in cases of malignancy and infection, with inconclusive bronchoscopic results even if antibiotic therapy had been initiated prior to biopsy. This is higher than previously reported by Hur et al. who found that CT guided needle aspiration had a sensitivity, specificity, and accuracy of 84, 100 and 91% in patients after an indeterminate transbronchial biopsy [18]. This is also significantly higher than that reported in patients who underwent a second transbronchial biopsy after the initial indeterminate biopsy, in whom the sensitivity, specificity and accuracy was 50, 100 and 63% [18]. Several important differences in our technique likely explain our improved diagnostic accuracy. We utilize rapid onsite cytologic evaluation to determine the need for further needle aspiration or tissue cores. The co-axial technique allows us to perform multiple needle aspirations and core biopsies through a single pleural puncture.

CNB was performed in addition to FNA in 22 of 30 cases. In all cases where CNB made the diagnosis of malignancy, the FNA was also positive for malignancy. This low added diagnostic yield of CNB is contrary to previous reports. In a prospective study of 48 patients with pneumonia and pneumonia-mimics who underwent both FNA and CNB, a specific diagnosis was made by FNA in only 10/48 cases (21%) compared to 42/48 (88%) by CNB [10]. Our higher yield with FNA is likely related to rapid on-site evaluation of FNA smears allowing for assessment of the adequacy of the obtained sample. In cases without a positive onsite evaluation, needle repositioning and further needle aspirations improve accuracy of FNA.

With rapid advances in targeted cancer therapies, the role of PTNB for molecular testing of non-small cell lung cancer (NSCLC) continues to increase [19]. Several studies have found that PTNB provides sufficient tissue for molecular testing of NSCLC [20, 21]. Our study confirms that molecular testing can be safely performed when lung cancer presents as consolidation. We obtained sufficient samples for molecular testing in all 14 cases of lung cancer, including two cases when only FNA were obtained. The use of on-site cytology may have had a role in our high success rate, as it confirms a positive site of tumor. This is especially important in patients with positive mutations who undergo repeat biopsy on development of resistance to targeted therapy, as there may be areas of partially treated cancer. The identification of viable tumor cells on rapid-cytologic analysis is likely to reflect a sufficient sample for molecular testing.

We found PTNB to be a safe technique to sample consolidations. Previous studies of PTNB of pulmonary lesions have reported 17–36.8% incidence of pneumothorax and 1–14.2% incidence of chest tube placement [4–6, 15, 22, 23]. Prior reported incidence of pneumothorax after PTNB of pulmonary consolidation was 8.3–48%; the lower figure is based on post procedural chest radiograph, and the higher figure is based on post procedural CT [10, 11]. The incidence of chest tube insertion in this cohort was up to 8.7% [10, 11]. In our study, the incidence of pneumothorax detected by chest radiograph was 10% and the incidence of chest tube insertion was 3%, which are within the previously reported ranges. There were two cases of mild hemoptysis in our study (7%), which is within the reported incidence (range, 0.2–8.4%) [4–6, 23, 24].

This study has several limitations. The retrospective nature of the study may have introduced a selection bias. Another limitation is the small number of cases included, a reflection of the infrequency at which PTNB is performed for evaluation of persistent consolidation. Our results are based on the experience at a single academic medical center and may not be widely applicable to centers that are not as experienced in PTNB or where on-site cytopathology is not available.

## Conclusion

In conclusion, pulmonary consolidation can be safely and effectively evaluated with CT-guided percutaneous needle biopsy. Malignancy can be accurately diagnosed and adequate tissue sampling can be performed for molecular testing when lung cancer or metastatic disease presents as consolidation rather than a nodule or mass. In infection, causative organisms can be identified after non-diagnostic bronchoscopy or prior antibiotic administration. A negative PTNB result is more likely to occur with an underlying benign diagnosis. Our results support the important diagnostic role of PTNB in the evaluation of persistent pulmonary consolidation, particularly in cases where prior bronchoscopic evaluation is nondiagnostic.

## Abbreviations

CLL: Chronic lymphocytic leukemia
CNB: Core needle biopsy
CT: Computed tomography
FNA: Fine needle aspirate
IRB: Institutional review board
MAI: Mycobacterium avium intracellulare
MDCT: Multidetector CT
NSCLC: Non-small cell lung cancer
PTNB: Percutaneous transthoracic needle biopsy
SD: Standard deviation
